# Full-duplex bidirectional data transmission link using twisted lights multiplexing over 1.1-km orbital angular momentum fiber

**DOI:** 10.1038/srep38181

**Published:** 2016-11-30

**Authors:** Shi Chen, Jun Liu, Yifan Zhao, Long Zhu, Andong Wang, Shuhui Li, Jing Du, Cheng Du, Qi Mo, Jian Wang

**Affiliations:** 1 Wuhan National Laboratory for Optoelectronics, School of Optical and Electronic Information, Huazhong University of Science and Technology, Wuhan 430074, Hubei, China; 2 Fiberhome Telecommunication Technologies Co. Ltd, Wuhan 430074, Hubei, China

## Abstract

We present a full-duplex bidirectional data transmission link using twisted lights multiplexing over 1.1-km orbital angular momentum (OAM) fiber. OAM_+1_ and OAM_−1_ modes carrying 20-Gbit/s quadrature phase-shift keying (QPSK) signals are employed in the downlink and uplink transmission experiments. The observed mode crosstalks are less than −15.2 dB, and the full-duplex crosstalks are less than −12.7 dB. The measured full-duplex optical signal-to-noise ratio (OSNR) penalties at a bit-error rate (BER) of 2 × 10^−3^ are ~2.4 dB in the downlink transmission and ~2.3 dB in the uplink transmission. The obtained results show favorable full-duplex twisted lights multiplexing data transmission performance in a km-scale OAM fiber link.

The last two decades saw dramatic expansion both in system capacity and data traffic^1^,^2^. Ever increasing research efforts for sustainable increase of transmission capacity have been devoted to overcome the emerging capacity crunch. In addition to traditional solutions based on different advanced multi-level modulation formats such as m-ary phase-shift keying (m-PSK) and m-ary quadrature amplitude modulation (m-QAM) as well as various multiplexing techniques such as wavelength-division multiplexing (WDM), orthogonal frequency-division multiplexing (OFDM), time-division multiplexing (TDM) and polarization-division multiplexing (PDM), space-division multiplexing (SDM) has recently attracted great attention as a promising technology to further improve the transmission capacity and spectral efficiency^3^,^4^,^5^,^6^. SDM using few-mode fiber (FMF), multi-mode fiber (MMF), multi-core fiber (MCF) and few-mode multi-core fiber (FM-MCF) has been widely studied in fiber optical transmission systems showing impressive performance^6^,^7^,^8^,^9^. Very recently, SDM employing twisted lights^10^, also known as orbital angular momentum (OAM) carrying lights, provides an alternative approach to increasing the transmission capacity and spectral efficiency of optical communications. Similar to other mode sets in free space or fiber, twisted lights carrying OAM are another mode set with which to represent spatial modes. One can use different mode sets for SDM, and so does twisted lights carrying OAM. Twisted light is characterized by a helical phase front of exp(*ilφ*), possessing an OAM of 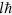 per photon, where the twist rate *l* is the topological charge number and *φ* is the azimuthal angle. It is noted that, in principle, *l* can take arbitrary integer number ranging from −∞ to ∞, and twisted lights carrying different OAM values are intrinsically orthogonal and separable with each other^11^. Thus OAM-division multiplexing (ODM), i.e. twisted lights multiplexing, as an alternative multiplexing technique of SDM, provides another potential way to enable the continuous increase of transmission capacity and spectral efficiency. So far there have been lots of significant research efforts to promote the transmission capacity and spectral efficiency both in free-space and fiber optical communication systems by employing twisted lights multiplexing, combined with WDM, PDM and advanced multi-level modulation formats^10^,^12^,^13^,^14^,^15^,^16^,^17^,^18^,^19^,^20^,^21^.

Remarkably, in practical optical communication systems, not only the large transmission capacity and high spectral efficiency are desired, but also the efficient usage of transmission links is preferred. There have been lots of efforts to develop bidirectional transmission systems, such as passive optical network, fiber-radio network, fiber-to-the-home link and free-space communication link, which enable the transmission link and the resultant cost of the equipment to be shared between the two directions of traffic in a full-duplex communication link^22^,^23^,^24^,^25^,^26^. Previously, single-mode fiber (SMF) or free space is considered for uplink and downlink transmission between both ends of the architecture. So far there have been very few research efforts devoted to bidirectional transmission systems using twisted lights multiplexing in fiber. In this scenario, a laudable goal would be to develop a full-duplex bidirectional data transmission link by exploiting twisted lights multiplexing using an OAM fiber.

In this paper, we propose and experimentally demonstrate a full-duplex data transmission link using twisted lights multiplexing over 1.1-km OAM fiber. The downlink and uplink transmit OAM_+1_ and OAM_−1_ modes carrying 20-Gbit/s quadrature phase-shift keying (QPSK) signals, respectively. The obtained results show that the mode crosstalks are less than −15.2 dB for both downlink and uplink transmission, while the full-duplex crosstalks are less than −12.7 dB. The measured full-duplex optical signal-to-noise ratio (OSNR) penalties at a bit-error rate (BER) of 2 × 10^−3^ (enhanced forward-error correction (EFEC) threshold) are about 2.4 dB for downlink and 2.3 dB for uplink. The demonstrated full-duplex OAM multiplexing transmission in 1.1-km OAM fiber shows favorable operation performance.

## Results

### Concept of OAM-fiber based full-duplex architecture

**Figure 1:**
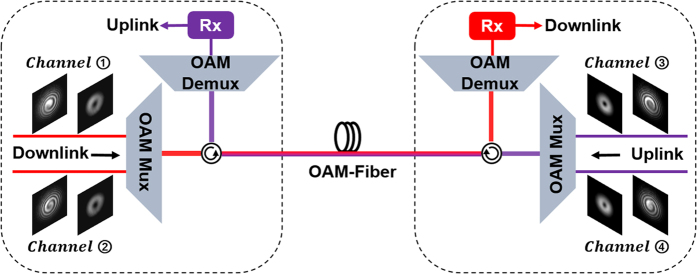
Concept of full-duplex bidirectional data transmission link using twisted lights multiplexing over 1.1-km OAM fiber.

Figure 1 shows the concept of full-duplex bidirectional data transmission link using twisted lights multiplexing over 1.1-km OAM fiber. In the downlink direction, two multiplexed twisted lights from channel ① and channel ② propagate through an OAM fiber, while the other two twisted lights from channel ③ and channel ④ in the uplink direction share the same transmission path. Thus these four bidirectional modes from four channels transmit in the same OAM fiber simultaneously. Note that all the four channels are orthogonal to each other by employing twisted lights with different OAM values and polarizations. One can also employ multiple OAM modes in the downlink and uplink directions to further increase the transmission capacity by employing OAM fiber supporting multiple OAM modes. After full-duplex bidirectional data transmission, the downlink and uplink twisted lights are separated from each other at the demodulation side and then sent to the receiver for followed offline processing.

### Experimental setup

**Figure 2:**
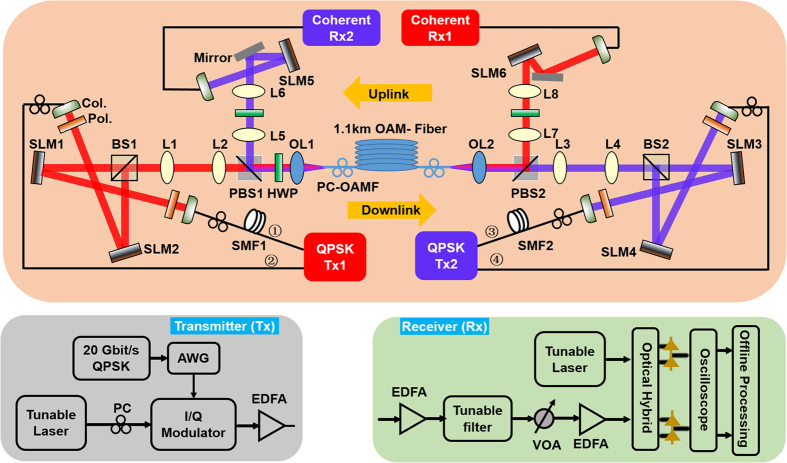
Experimental setup of full-duplex bidirectional data transmission link using twisted lights multiplexing over 1.1-km OAM fiber. QPSK: quadrature phase-shift keying; PC: polarization controller; AWG: arbitrary waveform generator; EDFA: erbium-doped fiber amplifier; SMF: single-mode fiber; Col.: collimator; Pol.: polarizer; SLM: spatial light modulator; BS: beam splitter; PBS: polarization beam splitter; HWP: half-wave plate; L: lens; OL: objective lens; PC-OAMF: polarization controller on OAM fiber; VOA: variable optical attenuator.

The experimental setup of full-duplex bidirectional data transmission link using twisted lights multiplexing over 1.1-km OAM fiber is shown in Fig. 2. At the transmitter side, an arbitrary waveform generator (Tektronix AWG 70002) is used to drive the IQ modulator generating 20 Gbit/s QPSK signal at a wavelength of 1550 nm. The signal is pre-amplified and split into four channels (channel ① and channel ② for downlink, channel ③ and channel ④ for uplink), and then relatively delayed by SMFs with different lengths for decorrelation. These four channels are launched onto four Holoeye PLUTO phase-only liquid crystal spatial light modulators (SLM1 and SLM2 for downlink, SLM3 and SLM4 for uplink) which are loaded with four hologram phase masks to create four OAM beams with topological charge of *l* = −1. The employed SLMs are polarization sensitive, i.e. working only for the x-polarization while having no response to the y-polarization. Thus the four generated OAM beams are initially at x-polarization (xOAM_−1_). Then we use a beam splitter (BS) to combine the two OAM beams together and expand the beams by lens pairs both for downlink and uplink. Note that each reflection can flip the topological charge sign of the OAM beam. So the combined OAM beams are OAM_+1_ and OAM_−1_, respectively. Before coupling into the 1.1-km OAM fiber by a 10X objective lens, the OAM beams for downlink are converted to y-polarization from x-polarization by a half-wave plate (HWP) while the uplink beams stay x-polarization. As a consequence, the OAM beams in the downlink direction are yOAM_−1_ for channel ① and yOAM_+1_ for channel ②, while in the uplink direction are xOAM_−1_ for channel ③ and xOAM_+1_ for channel ④, respectively. After transmission through the 1.1-km OAM fiber, the OAM beams are collimated by another 10X objective lens. Then the uplink x-polarization beams are converted to y-polarization by the HWP and reflected by the polarization beam splitter (PBS), while the downlink beams stay y-polarization and are reflected by another PBS. Here the PBS works like an optical polarization circulator as the x-polarization OAM beams transmit through the PBS (uplink before PBS2 and downlink before PBS1), while the y-polarization OAM beams are reflected by the PBS (uplink before PBS1 and downlink before PBS2) to the demodulation side. The four output OAM beams after full-duplex bidirectional transmission are shrunk by lens pairs and projected to another SLMs (SLM6 for downlink, SLM5 for uplink) for demultiplexing/demodulation. The demodulated Gaussian-like beam is followed by coherent detection at the receiver. In the OAM-fiber based full-duplex experiment, y-polarization for downlink and x-polarization for uplink through the OAM fiber are adopted to minimize the bidirectional crosstalk. Meanwhile, we adjust the polarization controllers on OAM fiber (PC-OAMF) to minimize the mode crosstalk and achieve desired output OAM modes with high quality.

## Experimental Results

**Figure 3:**
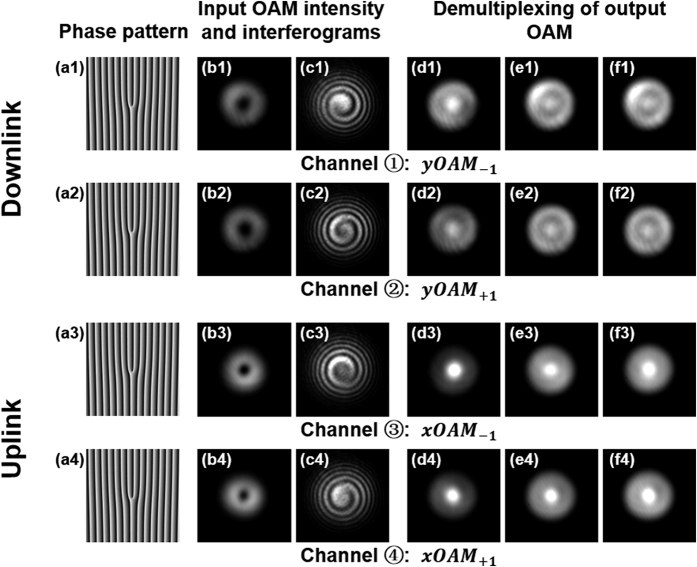
(**a1–a4**) Hologram phase masks loaded to SLM1, SLM2, SLM3 and SLM4 for generating four OAM beams with topological charge *l* = −1. (**b1–b4**) Observed input OAM intensity profiles and (**c1–c4**) interferograms with single channel ①, ②, ③ and ④ on. Observed demultiplexing of output OAM modes with (**d1–d4**) single channel on, (**e1–e4**) double channels on, and (**f1–f4**) all four channels on.

We first study the performance over the 1.1-km OAM fiber transmission for both downlink and uplink. The hologram phase masks^10^,^12^ loaded to SLM1, SLM2, SLM3 and SLM4 for generating four OAM modes with topological charge *l* = −1 are showed in Fig. 3(a1)–(a4). The followed reflections by BS and HWP enable the generation of four OAM modes, i.e. xOAM_+1_, xOAM_−1_, yOAM_+1_ and yOAM_−1._ For the downlink transmission, Fig. 3(b1) and (b2) show the observed intensity profiles of the generated input OAM modes when only channel ① or channel ② is on, respectively. The interferograms of the input OAM modes are obtained by interfering OAM mode with a reference Gaussian beam with the same polarization, as shown in Fig. 3(c1) and (c2). According to the number of twist and the twist direction, one can determine the topological charge number of OAM mode to be −1 or +1. At the demodulation side, hologram phase mask with corresponding inverse OAM charge number is loaded to SLM6. Thus the OAM beam is converted to Gaussian-like beam with a bright spot at the beam center. The obtained intensity profiles of the output OAM modes after demultiplexing with single channel on (only channel ① or channel ② on) are shown in Fig. 3(d1) and (d2). Moreover, Fig. 3(e1) and (e2) show the demultiplexing intensity profiles with both channel ① and channel ② on, while the demultiplexing intensity profiles with all four channels ①–④ on are shown in Fig. 3(f1) and (f2). Similarly, for the uplink transmission, the intensity profiles and interferograms of the input OAM modes in channel ③ and channel ④ are shown in Fig. 3(b3). The observed demultiplexing intensity profiles of output OAM modes with single channel on, double channels on and all four channels on are displayed in Fig. 3(d3), respectively.

**Figure 4:**
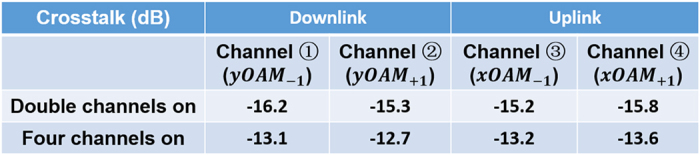
Recorded crosstalks with double channels on and all four channels on for downlink and uplink transmission.

Figure 4 records the crosstalks with double channels on and all four channels on for downlink and uplink transmission. Taking channel ① as an example, the crosstalk for channel ① with double channels on is exactly the mode crosstalk between channel ① and channel ② when channel ③ and channel ④ are off. Similarly, in the case of full-duplex when all four channels are on, the crosstalk for channel ① include both mode crosstalk and bidirectional crosstalk. One can see that the mode crosstalks are less than −15.2 dB, and the bidirectional crosstalks are less than −12.7 dB.

**Figure 5:**
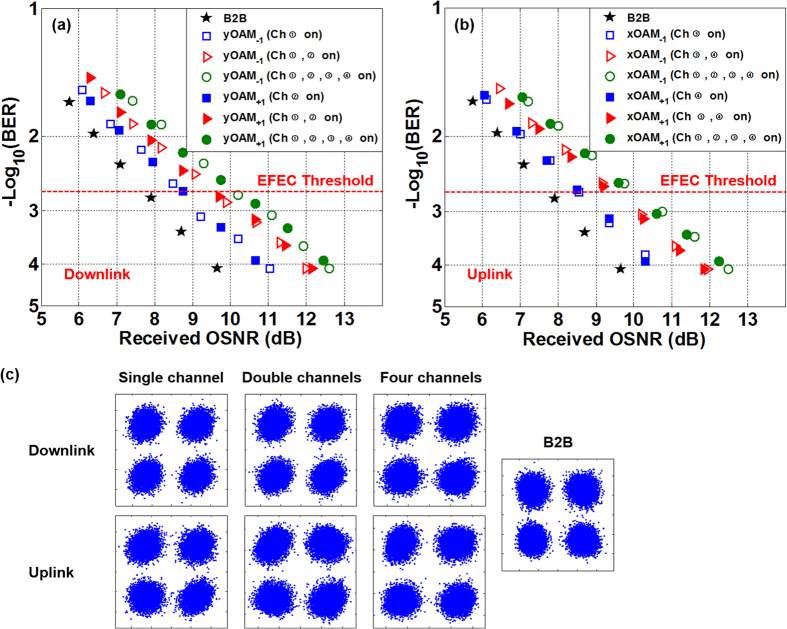
Measured BER versus received OSNR for (**a**) downlink and (**b**) uplink transmission over 1.1-km OAM fiber. (**c**) Received QPSK constellations with single channel on, double channels on, and all four channels on for downlink and uplink transmission. Back-to-back (B2B) QPSK constellation is also shown for reference.

We further measure the BER performance of full-duplex 20-Gbit/s QPSK transmission link using twisted lights multiplexing. Figure 5(a) and (b) plot the measured BER values as a functions of received OSNR for downlink and uplink transmission over 1.1-km OAM fiber, respectively. In the downlink transmission, compared to the back-to-back (B2B) case, the measured OSNR penalties at a BER of 2 × 10^−3^ (enhanced forward-error correction (EFEC) threshold) with single channel on, double channels on and all four channels on for two OAM modes (yOAM_−1_ and yOAM_+1_) are about 1 dB, 1.8 dB and 2.4 dB, respectively. Similarly, in the uplink transmission, the measured OSNR penalties at a BER of 2 × 10^−3^ with single channel on, double channels on and all four channels on for two OAM modes (xOAM_−1_ and xOAM_+1_) are about 0.8 dB, 1.6 dB and 2.3 dB, respectively. The received 20-Gbit/s QPSK signal constellations with single channel on, double channels on, and all four channels on for downlink and uplink transmission at a BER of ~1 × 10^−4^ are shown in Fig. 5(c). The back-to-back QPSK constellation is also shown for reference. According to the obtained results shown in Figs 3, 4 and 5 one can clearly see that the full-duplex data transmission link using twisted lights multiplexing over 1.1-km OAM fiber is successfully demonstrated in the experiment with a favourable transmission performance.

## Discussions

In summary, we report a full-duplex data transmission link using twisted lights multiplexing over 1.1-km OAM fiber. We employ OAM_+1_ and OAM_−1_ modes carrying 20-Gbit/s QPSK signals in the uplink and downlink transmission. The measured mode crosstalks between OAM_+1_ and OAM_−1_ modes are less than −15.2 dB, and the bidirectional crosstalks in the full-duplex link are less than −12.7 dB. At a BER of 2 × 10^−3^, the measured OSNR penalties with single channel on, double channels on and all four channels on for OAM_+1_ and OAM_−1_ modes are about 1 dB, 1.8 dB and 2.4 dB in the downlink transmission, and about 0.8 dB, 1.6 dB and 2.3 dB in the uplink transmission. The obtained results indicate favourable transmission performance of the full-duplex link based on twisted lights multiplexing over 1.1-km OAM fiber.

### Crosstalk

Remarkably, the mechanisms for the generation of crosstalk include two parts. One is the mode crosstalk between the two multiplexing modes along the same direction data transmission, while the other is the bidirectional crosstalk between the downlink and uplink directions. In the experiments, the measured mode crosstalk less than −15.2 dB and bidirectional crosstalk less than −12.7 dB are relatively high. With future improvement, the mode crosstalk could be improved by employing other specialty fiber structures such as high-index ring fiber designed to increase the effective refractive difference among different OAM modes (>10^−4^)^15^. The bidirectional crosstalk might come from the reflection on the fiber facet, which could be improved by making an angled fiber facet.

### Scalability

In the experiments, the employed 1.1-km OAM fiber can only support OAM_+1_ and OAM_−1_ modes. The main limitations in expanding the system for multi-OAM modes or higher-order OAM modes are specialty fiber structures supporting multi-OAM modes. Fortunately, different kinds of specialty fiber designs have been proposed and even fabricated to support multi-OAM modes with low-level mode crosstalk such as 12 high-order OAM modes in an air core fiber^27^, 22 modes with 18 OAM ones in a trench-assisted multi-OAM multi-ring fiber^17^, and 36 OAM modes spanning 9 OAM orders supported in an annular fiber^28^. In this scenario, one could use these multi-OAM fibers with low-level mode crosstalk to further expand the system for multi-OAM modes or higher-order OAM modes.

## Methods

**Figure 6:**
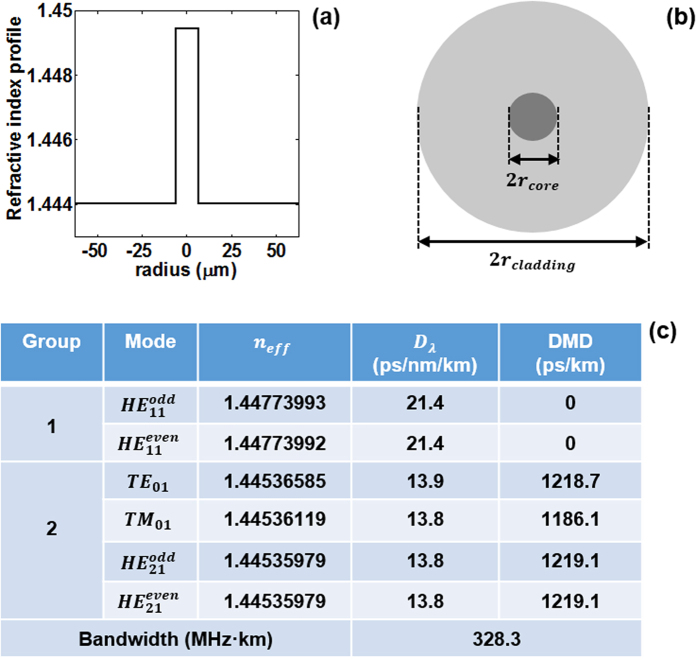
(**a**) Refractive index profile. (**b**) Cross-section view. (**c**) Mode properties supported in the OAM fiber. n_eff_: effective modal index; *D*_*λ*_: chromatic dispersion coefficient; DMD: delay between HE_11_ and other higher-order modes, respectively.

The employed 1.1-km OAM fiber in the experiment supports six eigenmodes in total (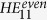, 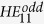, 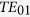, 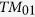, 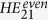,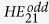). Figure 6(a) shows the refractive index profile of the OAM fiber. The diameters of the fiber core and cladding are 2r_core_ = 12.7 μm and 2r_cladding_ = 125 μm, respectively. The refractive index of the pure-SiO_2_ cladding and GeO_2_-doped core are 1.444 and 1.449 at 1550 nm, respectively. The cross-section view is shown in Fig. 6(b). We further evaluate the mode properties of the OAM fiber, including effective modal index (n_eff_), chromatic dispersion coefficient (*D*_*λ*_), differential mode delay (DMD) and bandwidth, as shown in Fig. 6(c). It is noted that xOAM_+1_, xOAM_−1_, yOAM_+1_ and yOAM_−1_ can be obtained by proper linear combinations of *TE*_01_, *TM*_01_, 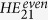 and 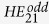.

## Additional Information

**How to cite this article**: Chen, S. *et al*. Full-duplex bidirectional data transmission link using twisted lights multiplexing over 1.1-km orbital angular momentum fiber. *Sci. Rep.* **6**, 38181; doi: 10.1038/srep38181 (2016).

**Publisher's note:** Springer Nature remains neutral with regard to jurisdictional claims in published maps and institutional affiliations.
